# Topology Reconfiguration for NoCs: A Fast Reconfiguration Algorithm Based on Monotonic Path Shifting

**DOI:** 10.3390/mi17040438

**Published:** 2026-03-31

**Authors:** Mingzhi Zhang, Zhijia Wang, Zhenxing Wang, Dali Xu, Na Niu

**Affiliations:** College of Computer and Control Engineering, Northeast Forestry University, Harbin 150040, China

**Keywords:** network-on-chip, topological reconstruction, core-level redundancy, REmesh, monotonic path shift

## Abstract

With the advancement of semiconductor technology, the Network-on-Chip (NoC) has become a critical architecture for communication between multiple cores. However, failures caused by factors such as manufacturing processes can degrade its performance and stability, making efficient topology reconstruction algorithms particularly important. Conventional 2D mesh reconstruction yields irregular topologies, increasing network latency and complicating system scheduling and deployment. While REmesh structures maintain topological regularity, they struggle to balance algorithmic complexity, success rates, and reconstruction costs. This paper proposes a monotonic path shift (MPS) topological reconstruction algorithm for REmesh NoCs with core-level redundancy, based on local rapid recovery. This algorithm localizes reconstruction decisions by establishing monotonic paths between failed cores and redundant cores for recovery. It incorporates region retention and local fallback mechanisms to suppress path conflicts among multiple failed cores. Theoretical analysis shows that MPS provides an upper bound on the runtime of the algorithm, significantly reducing its time complexity. Experimental results indicate that its reconstruction success rate is comparable to that of the ACTR algorithm, with both maintaining a high repair rate even under high fault density. In terms of core reuse rate, MPS achieves significant improvements over BTTR, BSTR, and ACTR, with an average increase of approximately 10% under low-fault conditions, effectively utilizing remaining computational resources. Concurrently, the algorithm substantially reduces average migration time, accelerating recovery by several orders of magnitude in large-scale low-fault scenarios and markedly lowering online recovery overhead.

## 1. Introduction

Very large scale integration (VLSI) technology continues to evolve under the impetus of Moore’s Law, enabling exponential growth in the number of transistors and functional modules integrable onto a single chip [1]. The synergistic gains in performance and power efficiency achieved through Dennard scaling in early stages gradually diminished beyond deep submicron processes, presenting severe challenges in overcoming the power wall and sustaining performance improvements [2,3]. Concurrently, computational demands for artificial intelligence, data centers, and edge computing have propelled SoCs toward multi-core/many-core architectures and heterogeneous accelerators [3,4]. Against this backdrop, on-chip global interconnects have increasingly become a system-level bottleneck: as process scaling progresses, the latency and energy consumption of fixed-length interconnects fail to decrease proportionally, significantly elevating the importance of interconnects in terms of latency, power consumption, and verifiability [5]. Consequently, adopting hierarchical, scalable Network-on-Chip (NoC) as the communication infrastructure has become the mainstream design paradigm for modern VLSI/SoC [6,7,8].

However, advanced processes have also significantly amplified reliability issues: manufacturing defects, process variations, aging effects (such as electromigration and BTI), and radiation-induced transient soft errors continuously increase the probability of circuits encountering permanent/transient failures throughout their entire lifecycle [9,10]. Within NoCs, failures in processing cores, routers, or links disrupt mesh connectivity, causing path interruptions, congestion, performance degradation, and even system unavailability [11]. Consequently, rapidly restoring a “topology-friendly” logical interconnect structure for upper-layer software after failures has become a critical focus in NoC reliability research [11].

To address the aforementioned issues, existing NoC fault-tolerance research typically proceeds on multiple levels. First, at the routing layer, researchers have proposed various fault-avoidance methods, such as fault-tolerant routing, adaptive detouring, and bufferless deflection. These methods bypass faulty nodes or links by dynamically adjusting packet forwarding paths without altering the underlying physical or logical topology, thereby offering advantages such as flexible deployment and rapid response [12]. However, these methods typically come at the cost of more complex routing control logic, longer communication paths, and potential latency jitter and increased congestion. Second, at the addressing and coordinate layers, some studies have introduced virtual coordinates or logical addressing mechanisms to decouple routing decisions from the physical location of failures, thereby enhancing the network’s recoverability under irregular failure conditions [13]. Furthermore, at the architectural level, by reserving spare cores and incorporating structural-level redundancy designs, computational and communication resources can be reorganized after a failure, thereby enhancing system robustness at the structural level [14]. Overall, NoC fault tolerance cannot be achieved through a single mechanism and often requires coordinated design across multiple layers—including routing, mapping, and topology—to balance performance loss, hardware overhead, and implementation complexity.

Among these methods, topology reconfiguration is widely regarded as one of the key strategies for addressing core-level failures [11]. The core idea of topology reconfiguration is to rebuild a logically interconnected network that remains available after a failure by adjusting the mapping relationships of computational resources and the logical connection structure of the network. This process typically involves operations such as data path rerouting, processing unit reassignment, and the reconstruction of logical adjacency relationships, thereby maintaining the regularity of the network structure as much as possible while avoiding the failed core or link. Unlike methods that rely solely on routing detours, topology reconfiguration focuses more on the global structural recovery following a failure, making it particularly suitable for scenarios with a high incidence of permanent failures or complex failure distributions.

Furthermore, research on topology reconstruction generally relies on core-level redundancy mechanisms [11,14]. This mechanism reserves a certain number of spare cores within the NoC system, allowing failed cores to be logically replaced, thereby ensuring system functional continuity and the availability of computational resources. When a failure occurs, topological reconfiguration algorithms can migrate tasks originally mapped to the failed core to a spare core while reorganizing the logical network structure to ensure that the reconfigured processing array retains a regular form as much as possible. This approach not only enhances the system’s fault tolerance but also maintains, to a certain extent, the consistency of higher-level programming models and communication abstractions, making it a significant research direction in NoC reliability design.

Meanwhile, with the advancement of reconfigurable computing, fault-tolerant and dynamically reconfigurable NoCs for FPGA hardware platforms have become a major area of research. Hosseinabady and Núñez-Yáñez were among the first to propose a fault-tolerant NoC-based SoC framework for dynamically reconfigurable systems, demonstrating the feasibility of combining runtime reconfiguration with fault avoidance in NoC architectures [15]. Subsequently, Beldachi designed a reconfigurable router for the SoCWire NoC, enabling the router architecture to work in concert with the FPGA’s dynamic reconfiguration process [16]; Hogan, meanwhile, built a NoC interconnect system on a partially reconfigurable FPGA platform, further validating the implementation foundation of local bitstream reloading and configuration readback to support online recovery [17]. In terms of soft error mitigation, Heiner proposed a method combining configuration scrubbing with partial reconfiguration, allowing the system to perform local reconfiguration while undergoing continuous scrubbing [18]; Cannon noted that the combination of TMR and configuration scrubbing is an effective means of mitigating radiation-induced soft errors in SRAM-based FPGAs [19]; Nguyen further investigated reconfiguration control networks for module-level error recovery in FPGA-TMR systems, demonstrating that relying solely on low-level reconfiguration channels is insufficient to guarantee overall reliability, as the effectiveness still depends on recovery control and system organization [20]. Therefore, such FPGA fault-tolerant frameworks that focus on low-level physical implementation mechanisms essentially still require collaborative design with higher-level algorithms—such as task mapping, routing reconfiguration, and resource reallocation—to determine efficient task migration paths and logical interconnections [21].

This paper focuses on designing an optimized topological reconfiguration algorithm that ensures the reconfigured topology remains as close as possible to the original structure while maintaining low time overhead and high resource utilization. Based on a two-dimensional REmesh topology, this study focuses on the topology reconstruction problem in on-chip networks (NoC). By proposing an optimized topology reconstruction algorithm, this paper aims to further optimize the recovery cost while maintaining the reconstruction success rate, thereby enhancing the algorithm’s applicability in larger-scale embedded systems.

The main contributions of this paper can be summarized as follows:

(1) We propose a local remapping reconstruction model constrained by monotonicity. Unlike existing methods that search for candidate replacement relations in the general remapping space [13,22], this paper reduces bad core recovery to the construction of UB/LB monotonic paths from the failed core to redundant columns and the problem of shifting mappings along these paths, and provides feasible update rules that satisfy the REmesh constraints.
(2) We propose a multi-fault conflict resolution mechanism that avoids global backtracking. To address path intersections and reachability blockages in multi-bad-core scenarios, we design a region-preservation and local-backtracking mechanism that adjusts the processing order within local conflict neighborhoods, thereby avoiding the expansion of conflict resolution into global combinatorial search.
(3) We evaluate algorithms based on recovery cost rather than merely their ability to find feasible solutions. Experiments across various scales and fault distributions demonstrate that MPS, while maintaining reconstruction success rates comparable to those of BSTR, BTTR, and ACTR, significantly improves core reuse rates and reduces average recovery time, with particularly pronounced advantages in large-scale, low-fault-rate scenarios [23,24,25].

The remainder of this paper is organized as follows: Section 2 briefly reviews relevant research progress. Section 3 introduces the REmesh structure and problem formulation; Section 4 presents the MPS method and algorithmic workflow; Section 5 details the experimental setup and results analysis; Section 6 concludes the paper and discusses future work.

## 2. Related Works

To address permanent processor core failures, incorporating redundancy techniques into the NoC is an effective fault-tolerance strategy. Adding redundant backup units to the architecture to replace failed units is currently a widely adopted technical approach. Depending on the level of redundancy, these techniques can be broadly categorized into two levels: microarchitectural-level redundancy and core-level redundancy [26,27].

Microstructure-level redundancy involves introducing redundant backup units or repairable structures for critical microstructural components within a processor core, enabling the core to maintain its original functionality even after a local device failure. The components targeted for repair in such approaches typically include critical modules such as register files, cache arrays, queues, execution units, and on-chip embedded memory [26,27]. Among these, memory built-in self-repair (MBISR) is a typical microstructure-level redundancy mechanism. It enhances memory reparability and chip yield by configuring redundant rows and columns within the memory array and utilizing techniques such as testing, analysis, and address remapping to replace faulty cells [26]. Overall, microarchitectural-level redundancy offers advantages such as fine-grained repair and the ability to preserve single-core functionality to the greatest extent possible. However, it also presents challenges including high design complexity, significant verification costs, and substantial variations in chip performance following repair [27]. In other words, even within the same batch of chips that have undergone redundancy-based replacements, their final usable performance may still vary significantly due to differences in fault locations and repair methods, which further increases the complexity of system tiering, scheduling, and deployment [27].

Compared to microstructure-level redundancy, core-level redundancy elevates the granularity of redundancy to the processor core level. Rather than focusing on repairing local components within a single core, it maintains system functional continuity by replacing failed cores with spare ones. Currently, core-level redundancy primarily includes two basic modes: the downgrade mode (As Many As Available, AMAA) and the redundancy mode (As Many As Demand, AMAD). The AMAA mode allows the processor to continue operating in a degraded state by masking the damaged core; the AMAD mode, also known as the N + M mechanism, consists of N functional cores and M redundant cores. When a core fails, it is replaced by a redundant core, thereby maintaining the normal operation of N available functional cores in the system [14]. Compared to microstructure-level redundancy, core-level redundancy offers greater flexibility in resource organization, task migration, and system-level recovery, making it more suitable for integration with mapping recovery, structural reconfiguration, and topological reconfiguration in NoCs [14].

As process feature sizes continue to shrink and advance to more advanced nodes, the impact of permanent defects on the yield and performance retention of multi-core/many-core chips has become increasingly significant. Research by Professor Premkishore Shivakumar and colleagues indicates that when feature sizes are smaller than 100 nm, core-level redundancy significantly outperforms microarchitecture-level redundancy in terms of the performance-weighted average yield metric Y_PAV [27]; subsequent research on homogeneous many-core systems further indicates that core-level redundancy is better suited for large-scale multi-core processors in terms of resource organization flexibility and system-level resilience [14]. Consequently, core-level redundancy has gradually emerged as a key technical approach for enhancing chip yield and performance.

Existing research on the reconstruction of core-level redundant NoCs can be broadly categorized into three types. The first category focuses on the abstraction of logical topologies following a failure. Rather than directly restoring physical interconnections, these methods redefine the mapping between logical and physical nodes to maintain a consistent view of the logical array for upper-layer software. The virtual topology concept proposed by Zhang et al. and the RRCS (Row Rippling and Column Stealing) method are representative examples of this category, with the core objective of reorganizing the logical array under fault constraints [13]. Around this objective, subsequent research has further shifted the optimization focus to the communication quality of the logical array. For example, by introducing distance factors (DF) and congestion factors (CF), and combining these with simulated annealing, shift-and-search improvements, and two-stage heuristic optimization, the impact of long paths and link congestion on communication performance has been mitigated [13,22,28]. Consequently, the focus of research in this direction lies not in whether the underlying physical structure remains unchanged, but rather in whether a usable, regular, and low-communication-cost logical topology can be provided to the system after a fault.

The second category of work focuses on resource handover and implementation support during the fault recovery process. In a core-level redundancy mode, the replacement of a failed core by a spare core is merely the starting point of the recovery process; subsequent steps still require addressing issues such as mapping updates, state migration, and connection control. Consequently, some studies have begun to address the placement of spare cores, fault injection, activation control, and recovery workflows from an implementation perspective, and have prototyped these mechanisms on FPGA platforms. For example, prior work has implemented hardware verification of fault-tolerant core mapping [29] and, following synthesis in the Vivado environment, verified the feasibility of adaptive core mapping on a Kintex-7 KC705 board [30]. Other studies have implemented spare core placement in a torus topology, runtime fault injection/activation mechanisms, and spare link configuration with table-driven fault-tolerant routing for ASNoC on similar platforms, further providing estimates of application runtime, communication overhead, resource consumption, and power consumption [31,32]. Such work demonstrates that core-level redundant NoC reconfiguration is not merely an algorithmic problem but also an engineering challenge closely tied to hardware synthesizability, control granularity, and online execution workflows. It also indicates that for reconfiguration algorithms to truly support runtime recovery, they must generate more explicit migration sequences and more localized replacement paths to form an executable closed-loop with debugging, control, and potential local reconfiguration mechanisms on the FPGA [29,30,31,32].

The third category of work focuses on topological recovery with physical consistency. Unlike methods that abstract the logical topology, this research posits that if logically adjacent nodes are physically separated after fault recovery, it will still introduce additional network latency and increase the complexity of system scheduling and application deployment. Therefore, the goal is no longer merely to restore a regular logical view, but to further restore the corresponding physical connection relationships. The reconfigurable two-dimensional REmesh architecture proposed by Wu et al. is based precisely on this approach. By incorporating additional spare columns, routers, and multiplexers, it enables the NoC to recover to a regular two-dimensional mesh even after a core failure, thereby advancing topology recovery from the logical layer to the level of physical consistency [33]. Within this framework, subsequent research has focused on balancing reconstruction success rates, search complexity, and recovery costs: BSTR improves the efficiency of finding feasible solutions through bidirectional search [23]; BTTR enhances dynamic reconstruction capabilities in complex failure scenarios via a backtracking mechanism [24]; and ACTR utilizes core distribution characteristics for adaptive allocation to improve the balance between reconstruction success rates and recovery time [25].

However, these topological restoration algorithms based on the REmesh structure also have the following limitations:

(1) The basic approach of the aforementioned methods still involves searching for feasible solutions within the global candidate mapping space. When selecting an adjacent healthy node to replace a failed node, this can easily trigger a chain reaction of healthy node replacements and result in the unnecessary consumption of redundant nodes;
(2) The computational complexity is high, making it difficult to strike a balance between topological reconstruction rate and topological recovery time;
(3) Most existing work focuses on obtaining “reconstruction results,” but lacks explicit descriptions of the information migration direction, migration path constraints, and migration consistency during the recovery process after reconstruction. This means that while the algorithms can complete topological repair, they cannot directly guide subsequent state migration and system recovery deployment.

Based on the above discussion, this paper proposes the monotonic path shifting (MPS) algorithm. This algorithm localizes decision-making to the construction of monotonic paths between faulty cores and spare columns, defines construction criteria (UB/LB) for monotonic paths, and constructs monotonic paths from faulty core nodes to redundant core columns. This approach clarifies the direction of information migration after reconstruction and reduces the use of unnecessary redundant cores. Additionally, this paper introduces a region-preservation mechanism to restrict the path search space and prevent entry into search dead zones. It also proposes a local rollback mechanism to mitigate reconstruction conflicts caused by incorrect search order of faulty cores in multi-faulty-core scenarios, thereby significantly reducing the average recovery time while maintaining a high success rate.

## 3. System Model and Problem Definition

### 3.1. REmesh Structure NoC Physical Topology

**Figure 1:**
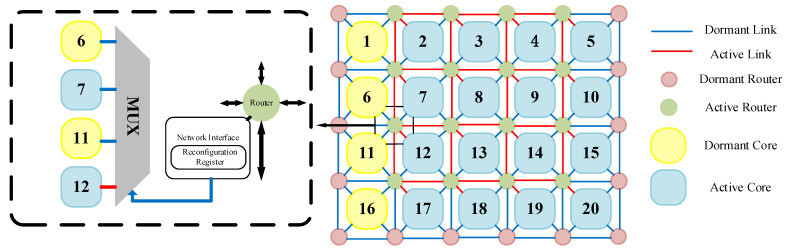
REmesh topology structure.

Figure 1 illustrates a typical 2D REmesh structure with a 4 × 4 working core grid, one redundant core column, and a 5 × 6 router network. This network primarily consists of on-chip routers, processor cores, network interfaces, links, and multiplexers. In this configuration, each processor core connects to the network via a router. Adjacent routers are linked by four-neighbor links, and the processor cores surrounding each router are interconnected through multiplexers. Except for components essential for maintaining system operation, all redundant components remain in a dormant state [33].

### 3.2. REmesh Structural Optimization and Logical Topology Reconstruction

**Figure 2:**
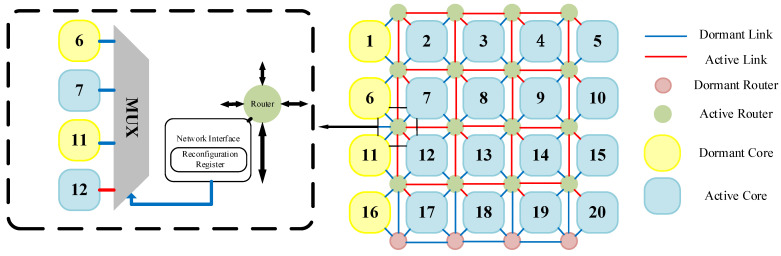
Optimized REmesh topology structure.

Although the REmesh structure offers good flexibility, it inevitably introduces excessive redundant routers. For the routers in the boundary columns, the topology of the 2D grid network within the N × N routing framework containing them degenerates into a standard 2D mesh. Therefore, we optimized the REmesh structure for the NoC. Figure 2 illustrates our optimized 4 × 5 REmesh structure. This configuration eliminates routers in the boundary columns, reducing both the area overhead caused by boundary routers and unnecessary search overhead during algorithm execution.

**Figure 3:**
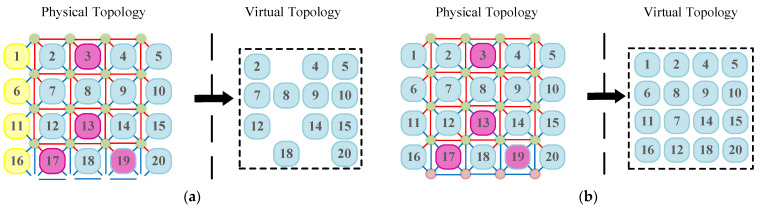
Topology restoration scheme for 2D REmesh structures: (**a**) permanent core failure occurs, (**b**) topology restoration scheme.

To provide a unified, regular programming view for operating systems and applications, it is necessary to construct a logical topology isomorphic to the target mesh when physical defect distributions change. The “virtual topology” concept proposed by Zhang et al. indicates that, with core-level redundancy support, logical nodes can be rebonded to available physical nodes through reconnection and mapping table updates, thereby shielding software from physical irregularities [13]. Within the REmesh architecture, the use of spare columns and multiplexers provides the hardware foundation for this transition from logical isomorphism to physical consistency. Figure 3a illustrates a permanent core failure in the original 2D mesh network topology. Leveraging the flexibility offered by the REmesh architecture, reconfiguring the multiplexers within the network enables the reconstruction of a 4 × 4 2D mesh network. Figure 3b illustrates a corresponding 2D grid reconstruction scheme. Evidently, the REmesh architecture not only mitigates the impact of faulty cores with robust fault tolerance, but also preserves the integrity of the 2D grid network topology.

### 3.3. Evaluation Indicators

Under the premise of meeting the constraints of connectivity and redundancy, the goal of mapping reconstruction is to comprehensively optimize communication performance and recovery cost in the feasible solution space. In order to facilitate the comparison of different methods, this paper employs the metrics proposed by Hou [25] to conduct a quantitative evaluation.

Successful Reconfiguration Rate (*SRR*): The proportion of the rule logic topology that can be successfully restored in random fault samples. This metric is the key to measuring the topology reconstruction algorithm, and the mathematical formula for the successful refactoring rate (*SRR*) of this experiment is as follows:

(1) $$SRR=\frac{N_{successful}}{N_{total}}\times 100\%$$

where $N_{successful}$ represents the number of successful topological reconstructions, and $N_{total}$ represents the total number of experimental tests.

Average Core Retention Rate (*ACRR*): The average proportion of cores that continue to function normally after reconstruction reflects the degree of disturbance to the original structure. The mathematical formula for the core reuse rate in this experiment is as follows:

(2) $$CRR=\frac{C_{reused}}{C_{total}}\times 100\%$$

(3) $$ACRR=\frac{CRR_{total}}{N_{successful}}\times 100\%$$

Among them, $C_{reused}$ represents the number of original cores that successfully completed the topological reconstruction, $C_{total}$ represents the total number of cores in the initial array, $CRR_{total}$ represents the sum of the kernel reuse rate of successful topology reconstruction, and $N_{successful}$ represents the number of successful refactorings.

Average Recovery Time (*ART*): Measures the average cost of mapping changes before and after successful reconstruction in random failure samples. The mathematical formula for mean time to recovery (*ART*) in this experiment is as follows:

(4) $$ART=\frac{T_{total}}{N_{successful}}$$

where $T_{total}$ represents the total running time of the topology reconstruction algorithm, and $N_{successful}$ represents the number of successful refactorings.

## 4. Map Reconstruction Algorithm Based on Monotonic Path Shift

### 4.1. Fundamental Concept of the Algorithm

The outcome of topological restoration is to utilize redundant cores, establishing a one-to-one correspondence between redundant cores and either defective or functional cores. In practice, only the mapping between defective cores and redundant cores is required, as the relationship between redundant cores and functional cores is unnecessary during the restoration process. Therefore, by starting from a defective core and identifying its most suitable redundant core, we can achieve the restoration of its topological structure.

To implement the MPS algorithm, we first introduce the concept of faulty core logical shifting: logical shifting involves disconnecting the switches of routers connected to faulty core units, thereby connecting to other functional cores within that router. The original routers connected to these functional cores are also disconnected, thus achieving logical shifting of the faulty core.

**Figure 4:**
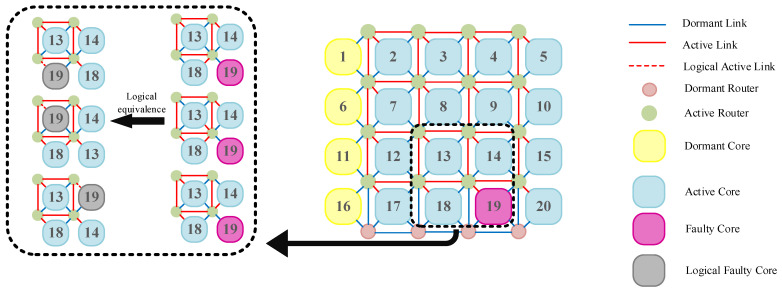
REmesh structural logic shift.

Figure 4 illustrates three methods for performing a single logical shift of a faulty core within a 4 × 5 REmesh structure. During this process, we disconnect core 19 from its connection to the upper-left router. Simultaneously, we select one of the functional cores connected to that router and disconnect that functional core from its connection to the upper-left router. This achieves the logical shift of the faulty core. The faulty core is then immediately connected to other functional cores linked to the faulty core’s router, effectively achieving a logical shift of the faulty core to the functional core it is now attached to.

For clarity, the main symbols used in the following formulation are summarized in Table 1. Use mappings to represent “logical cores hosted by physical cores.” Let the set of logical cores be L, with each physical core constrained to host at most one logical core (injectivity). Definition: m:V∖F→L is a mapping for each path $\pi_{i}=\left(p_{i,0},p_{i,1},\ldots ,\;p_{i,L}\right)$ with the update rule “move the mapping of $p_{i,t}$ to $p_{i,t-1}$”:$\;m^{\prime }\left(p_{i,t-1}\right)=\;m\left(p_{i,t}\right),\;t\;=\;1,\ldots ,\;L$. This process ultimately vacates the fault endpoint $p_{i,L}$ and occupies the spare column start point $p_{i,0}$, effectively shifting the faulty core logic to the redundant core. This operation corresponds to the completion of replacing the faulty core with the redundant core.

**Figure 5:**
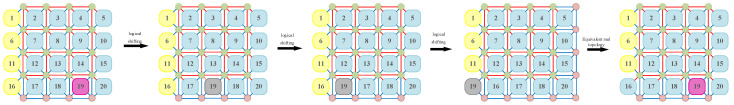
Implementation of REmesh structural logic mapping reconstruction.

Figure 5 illustrates how the 4 × 5 REmesh-structured NoC achieves topological restoration for a defective NoC using mapping rules. By applying these rules, we can construct a faulty-core path, $\pi_{1}=\;\left(16,\;17,\;18,\;19\right)$. The results demonstrate that this approach only requires locally reconstructing the corresponding path π. Starting from π, we disconnect the routers connected to processor cores 17, 18, and 19 along the path, then reconnect them to processor cores 16, 17, and 18 to achieve topology restoration. This eliminates unnecessary overhead associated with global reconstruction. Essentially, it involves shifting the faulty core to a redundant core through a finite number of logical shifts. Based on this principle, we can reduce the problem to finding a reachable path from the faulty core to the redundant core.

### 4.2. Monotonic Path Construction (Bidirectional)

**Figure 6:**
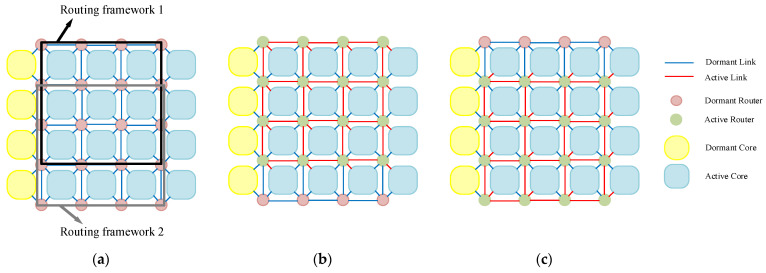
Selection of routing frameworks: (**a**) dual-routing framework hybrid topology, (**b**) routing framework 1 active state topology, (**c**) routing framework 2 active state topology.

For the optimized 4 × 5 2D REmesh structure, we have only two ways to select routing frameworks. Figure 6a illustrates both selection methods, while Figure 6b shows the connection pattern when selecting routing framework 1 without permanent core failures: the router defaults to using the processor core in its lower-right quadrant. Figure 6c illustrates the connection pattern when selecting routing framework 2, assuming no permanent core failures: the router defaults to using the processor core in its upper-right quadrant.

Depending on the selected routing framework, we can categorize path construction into two scenarios:

Case 1: When the upper routing framework is selected, if a permanent core failure occurs, the faulty core can only be supported by other normal processor cores connected to the router that initially enabled the faulty core. The permitted steps are ←, ↖, ↑.

Case 2: When the lower routing framework is selected: if a permanent core failure occurs, the faulty core can only be supported by other functional processor cores connected to the router that initially enabled the faulty core. The permitted steps are ←, ↙, ↓.

To reduce search complexity and migration overhead, we stipulate that in Scenario 1, the search direction prioritizes ←, followed by ↖, and finally ↑. This is termed the (Upper Base) UB criterion, and the constructed path is denoted as an upper-monotonic path. In Scenario 2, the search direction prioritizes ←, followed by ↙, and finally ↓. This is termed the (Lower Base) LB criterion, and the constructed path is denoted as a lower-monotonic path. If all three directions become unavailable, path construction is deemed unsuccessful.

### 4.3. Multi-Fault Handling: Sequence Adjustment and Conflict Avoidance

When the number of faults $\left|F\right|>\;1$, the shift paths for different faults may intersect or mutually block each other. Consider k fault nuclei $f_{1},\ldots ,f_{k}$, and select a monotonic path satisfying the constraints for each fault nucleus: $\pi_{i}=\;\left(p_{i,0},\;p_{i,1},\;\ldots ,\;p_{i,L_{i}}\right),\;p_{i,L_{i}}=\;f_{i},\;p_{i,0}\in \;\left\{\left(r,\;0\right)\right\}$. Denote the set of path points as: $V\left(\pi_{i}\right)=\;\left\{p_{i,0},\;p_{i,1},\;\ldots ,\;p_{i,L_{i}}\right\}$.

**Definition 1.** 
*(Path Families with Non-Overlapping Vertices): If for any* i ≠ j *,* $V\left(\pi_{i}\right)\cap \;V\left(\pi_{j}\right)=\emptyset$ *, then the path family* S *is said to be pairwise disjoint (vertices non-overlapping).*

**Theorem 1.** 
*Necessary and sufficient condition for conflict-free shifting: the vertices of the path family do not intersect.*

**Proof of Theorem 1** 
(Sufficiency)**.** If the vertices of the path families do not intersect pairwise, then the sets of physical cores involved in any two paths do not overlap. Updates on each path occur exclusively within their respective vertex sets $V\left(\pi_{i}\right)$. Consequently, the update scopes of multiple paths are mutually exclusive. Any physical core receives at most one update and at most one logical core, preventing conflicts where two paths write different logical cores to the same physical core. Furthermore, updates on a single path are transported sequentially without replication, ensuring the overall update maintains injectivity and $m^{\prime }$ is well-defined.

(Necessity) Suppose there exists an i ≠ j such that the two paths intersect, i.e., there exists a physical node v that belongs to the point sets of both paths simultaneously. We divide the discussion into two cases.

Case 1: If v appears as the “receiving location” in both paths during a step update, the overall update simultaneously requires: $m^{\prime }\left(v\right)=m\left(p_{i,t}\right)$ and $m^{\prime }\left(v\right)=m\left(p_{j,s}\right)$. This is equivalent to writing two (typically different) logical cores into the same physical core v, violating injectivity.

Case 2: If v appears as the “source location” in both paths and its predecessors differ ($p_{i,t-1}\neq \;p_{j,s-1}$), the update simultaneously requires sending the same carrier m(v) to two different locations: $m^{\prime }\left(p_{i,t-1}\right)=\;m\left(v\right)$, $m^{\prime }\left(p_{j,s-1}\right)=\;m\left(v\right)$. This is equivalent to copying the same logical core onto two physical cores, again violating injectivity. Both scenarios lead to contradictions, thus path families must have pairwise disjoint vertices. The theorem is proven. □

Due to the presence of multiple bad cores in the structure, no displacement conflicts can occur during topological restoration. As inferred from Theorem 1, we can reduce the problem to maximizing disjoint monotonic paths within the network.

For multiple failures, we first determine the processing order of faulty cores. For the upper routing framework, we select an initial search order from left to right and bottom to top. For the lower routing framework, we select an initial search order from left to right and top to bottom. This sorting strategy is critical for two reasons:

(1) Causality Preservation Mechanism: This mechanism ensures fault handling follows resource flow direction. By prioritizing nodes near array boundaries (or spare repositories), the algorithm clears reconstruction paths for subsequent upstream nodes, effectively preventing deadlocks and resource contention.
(2) Path Regularity: The geometric arrangement creates layered path flows. By paralleling shift chains in layers, it fundamentally reduces path crossing probabilities, transforming the global two-dimensional search complexity into a local one-dimensional traversal.

However, under the aforementioned initial construction sequence rules, since the construction path flows in a layered manner according to geometric arrangement, we can only determine that the sequence of faulty cores relative to the lower-left (including directly left and directly below) and upper-right (including directly right and directly above) regions is correct. To resolve conflicts caused by incorrect search sequences for faulty cores in other regions, we propose two types of mechanisms:

(1) Region Reservation Mechanism: Since the implementation of the region reservation mechanism is closely tied to the construction of monotonic paths, under multi-fault handling, the paths we construct flow in a layered manner according to geometric arrangement based on the search order. Therefore, for subsequent faulty core nodes, the search space does not need to traverse the space below this monotonic path. Otherwise, it would cause the monotonic path to enter a search dead zone, resulting in path search failure. Therefore, for the routing framework above, after each monotonic path is found, the region below this path is masked. For the routing framework below, after each monotonic path is found, the region above this path is masked.
(2) Local Backtracking Mechanism: During each search for monotonic paths, conflict detection is performed. If a conflict occurs, a local backtracking is executed by advancing the search order of the node causing the conflict. Since we have specified an initial search order, depending on the selected routing framework, conflict nodes only need to be sought within the upper-left/lower-left regions. The feasibility of this approach is demonstrated by Theorem 2 and 3 in the subsequent discussion.

**Definition 2.** 
*(Geometric Partial Order): Define a partial order *⪯ *on *F*: *$u⪯v\;⟺\;r_{u}<r_{v}\wedge \;c_{u}<c_{v}$*, meaning node u is located to the upper left of node v (excluding directly above and directly to the left).*

**Definition 3.** 
*(Reachability Cone and Blocking) Let* $G_{res}=V\setminus V_{occ}$ *denote the current residual graph. The reachability cone* $\Omega \left(f\right)$ *of node f is defined as the set of all paths originating from f in* $G_{res}$ *that satisfy the UB rule and reach a redundant column. If* $\Omega \left(f\right)=\emptyset$ *, node f is said to be blocked.*

**Definition 4.** 
*On a two-dimensional plane, any UB path π without self-loops divides the grid space V into two disconnected regions: the upper domain* $R_{above\left(\pi \right)}$ *, comprising nodes “above” the path, and the lower domain* $R_{below\left(\pi \right)}$ *, comprising nodes “below” the path.*

Properties: Since an UB path consists of left (←) and up (↑) components, for any $u\;\in \;R_{above\left(\pi \right)}$, all its UB paths $\pi_{u}$ leading to the reserve column must be entirely contained within $R_{above\left(\pi \right)}\cup \;\pi$ (unless traversing π); that is, UB paths form the “ceiling” boundary for nodes below them and the “floor” boundary for nodes above them.

**Theorem 2.** 
*When the above routing framework is selected: Consider two faulty nodes u, v ∈ F such that *u ⪯v *(*i.e.*,u is located to the upper-left of v). Let *$\Pi \left(u\to v\right)$ *denote the strategy of “planning a path for u first, then for v,” and *$\Pi \left(v\to u\right)$ *denotes the reverse, if u is a conflict node under policy *Π(v→u)*, then policy *Π(u→v)*can resolve the conflict caused by* Π(v→u).

**Proof of Theorem 2** 
(Since the proofs for Theorem 2 and Theorem 3 are similar, differing only in direction, only the proof for Theorem 2 is listed below)**.**

Step 1: Analyze potential conflicts in $\Pi \left(v\to u\right)$ (first address the lower-right node v)

Potential Conflict 1: If node v in the lower-right enters the search dead zone below u during pathfinding (resulting in v’s pathfinding failure), v becomes unreachable.

Potential Conflict 2: Suppose a path $\pi_{v}$ is first found for v. Per the UB rule, path $\pi_{v}$ must exhibit both leftward and upward tendencies. Based on Definition 4, whether u lies in $R_{above\left(\pi_{v}\right)}$ or $R_{below\left(\pi_{v}\right)}$, it becomes indeterminate.

If $\pi_{v}$ crosses “above” u, then: $\Omega \left(u\right)\cap \;R_{above\left(\pi_{v}\right)}=\;\emptyset$. As an upper-left node, u’s solution space is already severely constrained by the physical boundary r=0. Disturbance from $\pi_{v}$ easily renders u unreachable.

If $\pi_{v}$ crosses “below” u and blocks the path to c=0, then: $\Omega \left(u\right)\cap \;R_{below\left(\pi_{v}\right)}=\;\emptyset$, and simultaneously $\Omega \left(u\right)\cap \;R_{above\left(\pi_{v}\right)}=\;\emptyset$, rendering u unreachable.

Step 2: Analyze the non-conflicting nature of $\Pi \left(u\to v\right)$ (starting with the upper-left node u)

Assume we first find the path $\pi_{u}$ for u, at this point, u cannot potentially encounter conflict 2. Since u is an UB path and the target is c=0, the trajectory vector d of $\pi_{u}$ belongs to the set $\left\{\left(0,-1\right),\;\left(-1,-1\right),\;\left(-1,0\right)\right\}$. Note that v lies to the lower right of u ($r_{v}>\;r_{u},\;c_{v}>\;c_{u}$). This implies u’s path extends away from v’s region: $\pi_{u}$ moves upper-left while v is lower-right. Thus, $\pi_{u}$ must entirely lie above and to the left of v. By Definition 4, $\pi_{u}$ effectively becomes a new “lower bound” for v. As $\forall p\in \pi_{u},r_{p}<r_{u}<r_{v}\;\mathrm{and}\;c_{p}<c_{u}<c_{v}$, this implies that $\pi_{u}$ is highly unlikely to sever v’s path to alternative columns (since v’s path can be found above it). Combined with the region preservation mechanism, we prevent $\pi_{v}$ from re-entering the search dead zone below u, thereby eliminating potential conflict 1. The reachable domain $\Omega \left(v\right)$ is merely compressed by $\pi_{u}$’s lower bound, while its primary left/upper search space $R_{above\left(\pi_{u}\right)}$ remains open. If u also exhibits a potential conflict, local backtracking will continue. We can always eliminate the conflict involving $\pi_{u}$ through local backtracking, thus proving Theorem 2. □

Local Backtracking Mechanism for the Upper-Layer Routing Framework: When the MPS algorithm detects a failure of $\Pi \left(v\to u\right)$, the partial retreat mechanism forces a switch to Π(u→v). This effectively eliminates potential conflict 1 by defining u as a Class A partial retreat point, and eliminates potential conflict 2 by defining u as a Class B partial retreat point.

**Figure 7:**
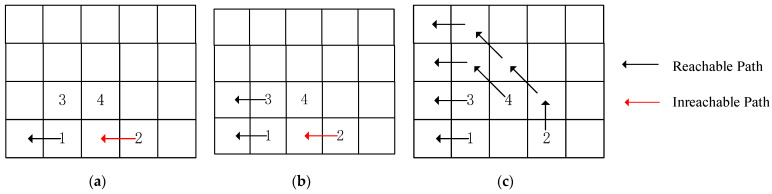
Local rollback mechanism for potential conflict scenario 1: (**a**) sequential search, (**b**) search after local backtracking, (**c**) globally reachable path derivation results.

Figure 7 illustrates a potential conflict 1 during monotonic path search in a 4 × 5 REmesh structure and the local backtracking strategy. Nodes 1, 2, 3, and 4 represent bad core nodes, with the initial path order constructed based on ascending numerical values. Figure 7a shows potential conflict 1 occurring when searching for the upward-monotonic path of the second bad core after completing the search for the first bad core. At this point, a Class A local backtracking point 4 is selected. Since 4 would block 3, another local backtracking occurs, prioritizing the search for 3. Figure 7b illustrates that, during the search for node 2, potential conflict 1 reappears. We prioritize searching for node 4. Due to the introduction of the region reservation mechanism, we perform region masking on the area below the searched path. This prevents node 2 from entering the search dead zone below node 4, allowing the monotonic path for node 2 to be successfully found, as shown in Figure 7.

**Figure 8:**
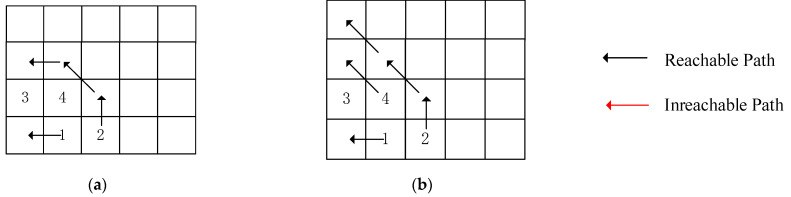
In potential conflict scenario 2, a local rollback mechanism above the conflict node resolves the conflict: (**a**) sequential search, (**b**) globally reachable path derivation results.

Figure 8 illustrates a potential conflict 2 occurring during monotonic path search in a 4 × 5 REmesh structure, showing the path traversing above the conflict node and the local backtracking strategy. Nodes 1, 2, 3, and 4 are bad core nodes, with the initial path order constructed based on ascending node numbers. Node 3 resides in a redundant column, where bad cores in redundant columns do not require search. Figure 8a illustrates that during sequential search, bad core node 2 restricts the reachability domain of bad core node 4, creating potential conflict 2. In this case, a Class B local retreat point 4 is selected, prioritizing the search for 4. Figure 8b shows that, by prioritizing the search for 4 followed by 2, the global monotonic path is successfully constructed.

**Figure 9:**
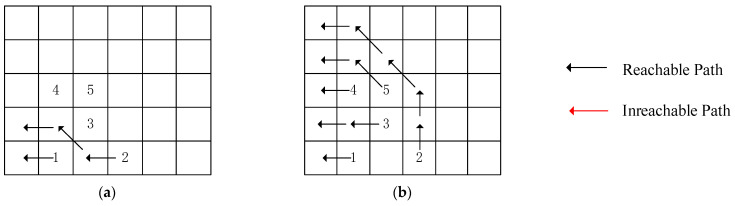
In potential conflict scenario 2, a local rollback mechanism below the conflict node resolves the conflict: (**a**) sequential search, (**b**) globally reachable path derivation results.

Figure 9 illustrates a potential conflict 2 occurring during monotonic path search in a 5 × 6 REmesh structure, where conflict node 2 traverses beneath another conflict node, along with the local backtracking strategy. Nodes 1, 2, 3, 4, and 5 represent bad core nodes, with the initial path order constructed based on ascending numerical values. Figure 9a illustrates a potential conflict 2 during sequential search: bad core node 2 traverses beneath node 3, restricting node 3′s reachable domain. A Class B local retreat point 3 is selected, prioritizing search for node 3. Figure 9b shows the subsequent application of multiple local retreats and region retention mechanisms, enabling successful construction of the global monotonic path.

**Theorem 3.** 
*When the routing framework below is selected: Consider two faulty nodes *u, v ∈ F *such that *u ⪯v* (*i.e.*,* *u is located below and to the left of v). Let  *$\Pi \left(u\to v\right)$* denote the strategy of “planning a path for u first, then for v,” and *Π(v→u) *denotes the reverse, if u is a conflict node under policy* Π(v→u)*, then policy* Π(u→v) *can resolve the conflict caused by *Π(v→u).

When BTTR triggers a search failure due to multiple faulty nodes, backtracking reverses the current router selection and blindly resumes searching for alternative solutions within the two-dimensional grid mechanism. Under high fault density, severe grid resource fragmentation causes this “reverse-and-rescan” action to cascade, forcing the algorithm to perform depth-first search (DFS) across all possible physical path combinations. Consequently, its worst-case complexity explodes exponentially. MPS introduces strict monotonic constraints (UB/LB). This reduces the search space for each failed node from a two-dimensional random walk to a directed layered flow. When MPS detects potential conflict 1 or potential conflict 2 across multiple nodes, it alters the “processing flowchart” for the failed nodes rather than changing the path generation rules. As proven by Reasoning 2 and Reasoning 3 in the paper, since paths are monotonic, the generated conflicts can be completely eliminated by simply swapping the processing queues of these two nodes (i.e., switching Class A/Class B rollback points), thereby avoiding the exponential trap.

### 4.4. Path Shifting

Once the path family S from the failed node to the standby column is identified, the mapping shift along the path can be executed: for each bad-core path $\pi_{i}=\;\left(p_{i,0},\;p_{i,1},\;\ldots ,\;p_{i,L}\right),\;i=1,2,\ldots ,k$, the virtual nodes originally mapped to $p_{i,t}$ are shifted to $p_{i,t-1}$, proceeding sequentially for t=L,L−1,…,1. After the shift completes, the failed position $p_{i,L}$ no longer carries any virtual nodes, and $p_{i,0}$ in the spare column becomes occupied, thereby replacing the failed node. The mapping shift along the path only requires disconnecting the routers connected to the processor cores on the path $\left(p_{i,1},\;p_{i,2},\;\ldots ,\;p_{i,L}\right)$ and reconnecting the processor cores connected to path $\left(p_{i,0},p_{i,1},\ldots ,p_{i,L-1}\right)$. Since the shift occurs only on local paths, its migration overhead is proportional to the path length, making it controllable and easily implementable online.

After reconstruction, existing routing-layer methods (such as detour or rerouting) are highly prone to introducing loop dependencies—and thus causing deadlocks—because they disrupt the regular path structure. In contrast, the MPS algorithm proposed in this paper, based on REmesh, performs reconstruction at the topological layer. At the physical layer, the MPS algorithm merely reconfigures multiplexers (MUXes) to alter the connection mappings between processors (Cores) and the local ports of routers (Routers). The underlying router-to-router network structure remains a perfect, regular 2D mesh with no disruption or addition to the order of routers [33]. Since the underlying router network remains a regular 2D mesh, the routing behavior of packets after reconstruction is entirely determined by the unchanged router network. Consequently, the system can continue to use classic, inherently deadlock-free deterministic routing algorithms (such as Dimension-Order XY routing) [34]. As packets are passed between routers, no new loop dependencies are created, thereby preventing deadlocks from occurring through both physical and logical mechanisms.

### 4.5. Algorithm Pseudocode and Complexity

Based on the above theory, this section innovatively proposes the monotone-path shifting (MPS) reconstruction algorithm. This algorithm aims to maximize the correspondence between bad cores and redundant cores, preventing unnecessary time overhead from global reconstruction through a local reconstruction approach. It further optimizes both the execution time complexity and the success rate of reconstruction via a local backtracking mechanism and a region retention mechanism. The main innovations of this algorithm are as follows:

(1) We propose a topology reconstruction algorithm based on local decision-making. During detection, this algorithm determines the direction for constructing monotonic paths to faulty cores based on the range of processor cores covered by the selected routing framework. Based on the constructed monotonic paths, we disconnect and reconnect processor cores on relevant routers along the paths to achieve path mapping shifts, ultimately reconstructing the topology. In this paper, we propose two approaches for selecting routing frameworks:

(a) Select the upper routing framework, where routers in this framework are connected by default to the processor core in the lower right.

(b) Select the lower routing framework, where routers in this framework are connected by default to the processor core in the upper right.

Based on the selected routing framework, we can define the initial search order for bad kernels and the construction direction for monotonic paths.

(2) The region reservation mechanism and local backtracking mechanism. The region reservation mechanism prevents subsequent path searches from entering dead ends by restricting portions of the search space after constructing monotonic paths. The local backtracking mechanism avoids potential conflicts arising during monotonic path construction through local backtracking, thereby significantly reducing both the algorithm’s execution time complexity and the success rate of algorithmic reconstruction.

The fundamental principles of MPS are as follows:

Step 1: Initialize the physical array H and search for the routing framework S within the physical array.

Step 2: Based on the selection of routing framework S, we initialize the search order for defective cores and the construction direction of monotonic paths.

Step 3: Perform path search for defective cores according to the order, utilizing the region reservation mechanism and local backtracking mechanism to restrict adjustments to the search order, and record the searched paths in the Path table.

Step 4: Perform shift mapping along paths based on the Path table.

Step 5: Generate the corresponding solution T under routing framework S.

To analyze the time complexity of the MPS algorithm, we illustrate using the example of target topology reconstruction on an n×(n+k) scale. In this algorithm, the routing framework must be traversed first. Therefore, in the best-case scenario, reconstruction succeeds under the first routing framework, resulting in a time complexity of O(1). In the worst-case scenario, all routing frameworks must be traversed, yielding a time complexity of O(2k). Thus, the average time complexity is O(k).

The overall procedure of the proposed MPS method is summarized in Algorithm 1.
**Algorithm 1.** MPS Algorithm.**Input:** An n × (n + k) physical array H **Output:** An n × n target array T1: **for ***i* = 0 to 1 **do** 2:      **for**
*j* = 0 to k−1 **do**
3:            Select a routing framework $S_{ij}$ 
4:            Select the n×(n+1) physical array $P_{temp}$ covered by the selected routing framework $S_{ij}$
5:            **if** *i* = 0 then /* The selected routing framework is the one above.*/ 6:                  T:=MPS_UB($S_{ij},P_{temp}$);
7:            else /* The selected routing framework is the one below.*/ 
8:                  T:=MPS_LB$S_{ij},P_{temp}$);
9:            **end if**
10:            **if** T is effective **then**
11:                  **Return** T
12:            **end if**
13:      **end for**
14: **end for**

In the process of constructing monotonic paths, the generated programs differ only in the order of the initial search for defect nuclei and the direction of path expansion. Here, we present only the upward search version, MPS_UB; see Algorithm 2 for the pseudocode.
**Algorithm 2.** MPS_UB Algorithm.**Input:** An n × n routing framework S, An n × (n + 1) physical array P, **Output:** An n × n target array T1: Initialize path table *Path*, path table copy ${Path}_{copy}$ 
2: Initialize the defective core linked list for the workspace Linklist /* Records defective cores in the workspace for constructing monotonic paths */
3: **for** i = n−1 to 0 **do //** Build the initial bad block search order from left to right and bottom to top
4:      **for** j = 0 to n **do**
5:            **if** $P_{ij}$ is a defective core **then**
6:                  Mark redundant column area bad blocks and mark bad blocks in the workspace to Path 
7:                  **if** j !=0 **then**
8:                        Update the bad core in the workspace to Linklist
9:                  **end if**
10:            **end if**
11:      **end for**
12: **end for**
13: ${Path}_{copy}:=Path$;
14: **while** Linkist is not empty **do**
15:      tag:=Find_UB_path(${Linklist}_{first},{Path}_{copy}$);// Construct a monotonic path π for ${\mathrm{for}\;Linklist}_{first}$ and record it in ${Path}_{copy}$, using tags to document the construction results.
16:      **if** tag **then**
17:            **if** Failed potential conflict 2 detection **then**
18:                  ${Path}_{copy}:=Path$;
19:                  Advance the selection of a Category B partial rollback point;
20:            **end if**
21:      **else**/* Potential conflict detected 1*/
22:            ${Path}_{copy}:=Path$;
23:            Advance the selection of a Class A partial rollback point;
24:      **end if**
25:      **if** No potential conflict 1 has occurred and No potential conflict 2 has occurred **then**
26:            $Path\;=\;{Path}_{copy}$;
27:            Perform region masking on Path  and ${Path}_{copy}$;
28:      **end if**
29: **end while**
30: Map and shift S and P along the path according to the Path table, and store the result in T;
31: **return** T

In terms of time complexity, determining the initial search order for bad cores and the first update of the Path table requires scanning the entire table, resulting in a time complexity of $O\left(n^{2}\right)$. The worst-case number of steps for a single path construction does not exceed $O\left(n\right)$ (moving at most N steps in the column direction and at most N steps in the row direction). Each fault attempts up to three directions, resulting in a basic cost of $O\left(\left|F\right|\cdot n\right)$ for searching all faulty cores. When including sequential adjustments, the worst-case scenario involves $O\left({\left|F\right|}^{2}\cdot n\right)$ reordering attempts. For local backtracking, the Path_copy table must be restored. The basic cost per backtracking step is $O\left(n^{2}\right)$. In the worst case, ${\left|F\right|}^{2}$ reordering attempts are required, resulting in a cost of $O\left({\left|F\right|}^{2}\cdot n^{2}\right)$. Additionally, updating the Path table incurs a cost of $O\left(\left|F\right|\cdot n^{2}\right)$. Therefore, the worst-case time complexity of the MPS_UB algorithm is $O\left({\left|F\right|}^{2}\cdot n^{2}\right)$.

Based on the preceding analysis of routing framework selection and the time complexity of the MPS_UB algorithm, the worst-case time complexity of MPS is $O\left(k\cdot {\left|F\right|}^{2}\cdot n^{2}\right)$. This provides a strict upper bound for the algorithm’s worst-case runtime.

## 5. Experiment

### 5.1. Experimental Preparation

To verify the algorithmic feasibility and performance of the MPS algorithm, this paper adopts the experimental scheme proposed by Hou [25]. A stimulation module is used to simulate the operational state of the entire network-on-chip (NoC) architecture, while a detection module filters non-redundant data from a massive amount of randomly generated data, providing a benchmark for evaluating the algorithm’s success rate on real-world data. Additionally, an independent sample’s t-test is employed to ensure a rigorous evaluation of the algorithm’s effectiveness in reconstructing the core NoC architecture.

This study constructed square network topology instances of various scales—including 4 × 5, 8 × 9, 12 × 13, and 16 × 17—as well as non-square network topology instances of 4 × 9 and 8 × 11 on 2D REmesh and 2D mesh architectures, with a focus on experiments involving specific designs containing redundant core columns. This design places the redundant column on the far left of the network. We systematically tested its core fault tolerance capabilities and simulated varying numbers of failed cores, ranging from one to the total number of redundant cores. During the experiments, for each configuration of failed cores within the same network scale, we performed 1000 random fault injections, using a stimulus generation module to simulate core failure scenarios during NoC operation. To further validate the effectiveness of the system’s redundancy design, we randomly injected 100,000 initial states into REmesh NoCs of varying scales and fault core counts, and used a detection module to evaluate their redundancy performance.

To highlight the feasibility and performance of the local reconfiguration strategy, this experiment focused on comparing: reconfiguration success rate (*SRR*), average core retention/reuse rate (*ACRR*) under different failure counts, the average recovery time (*ART*) under various failure scenarios, and the algorithm execution time (*AET*). Here, *ART* represents the average time required for the NoC to perform data migration after the algorithm generates a feasible reconstruction plan. Since, in practice, data migration time primarily depends on the time taken for processor cores to receive and process information, to simulate the latency caused by data migration in real-world scenarios, we do not account for the time spent on data transmission and assume that the time required for data migration between cores is 10 ns. The algorithm execution time refers to the time taken to execute a test case and generate the corresponding reconstruction scheme, which is used to measure the difference in time complexity between different algorithms.

In this paper, the algorithms are implemented in C and tested on a computer equipped with a 3.20 GHz CPU and 16 GB of memory. All algorithms in the experimental section are tested under a unified fault model, i.e., a physical topology with randomly distributed processing elements (PEs) is generated using a unified random generator. This method ensures the robustness and reliability of the experimental results.

### 5.2. Comparison Algorithms

This paper selects two categories of methods for comparative analysis, with the specific setup as follows:

The first category consists of primary benchmark methods, all of which are native 2D REmesh topological reconstruction algorithms. These include BTTR [24], which is based on a backtracking strategy; BSTR [23], which is based on a bidirectional search mechanism; and ACTR [25], which is based on adaptive optimization using core distribution. These methods share the same architectural assumptions and redundant resource organization paradigm as the proposed scheme in this paper, serving as the primary comparison targets. Through quantitative comparison with these methods, the performance improvements of the proposed MPS scheme under identical problem settings can be directly verified.

The second category consists of external supplementary benchmark methods, specifically RRCS and RRCS-gSA [13]. It should be explicitly noted that the original research scenarios of RRCS and RRCS-gSA focused on conventional 2D mesh topological reconstruction, whereas the subject of this study is the 2D REmesh logical topology recovery problem with redundant columns. The two approaches exhibit fundamental differences in core dimensions such as architectural assumptions and reconstruction objective functions. Therefore, this paper positions RRCS and RRCS-gSA as external reference benchmarks rather than primary benchmarks on the same level as the 2D REmesh native algorithm. To ensure fairness in the comparison, RRCS and RRCS-gSA were adapted and reimplemented under the following unified conditions: workspace size, redundancy budget constraints, failure model settings, and recovery cost models.

Under the unified adaptation framework of this paper, considering that the reconstruction algorithms of RRCS and RRCS-gSA are fundamentally designed for irregular topological scenarios, their natively defined distance parameter (DF) and congestion parameter (CF) lack effectiveness for the regular 2D REmesh structure. Therefore, they were not included in the comparison metric system; additionally, the reconstruction success rate of such methods primarily depends on the numerical relationship between the number of faulty nodes and the number of redundant nodes. Therefore, this paper focuses on comparing them from two core dimensions: recovery disturbance and recovery cost. Furthermore, the core mechanism of RRCS-gSA is to further optimize the mapping relationship between the virtual topology and the physical topology based on the feasible initial solution generated by RRCS, without introducing new active or redundant nodes. Consequently, the two methods exhibit consistent performance in terms of average recovery reconstruction rate (*ACRR*) and average recovery time (*ART*), with the primary performance difference manifesting in average end-to-end delay (*AET*).

### 5.3. Results and Analysis

(1) Success Rate of Reconstruction (*SRR*/Yield):

Since reconstruction algorithms for 2D mesh structures produce irregular reconstructions, the 2D mesh reconstruction can always yield a valid virtual topology when the number of faulty cores is less than the number of redundant cores, thereby ensuring the success of the topological reconstruction [13]. Therefore, in terms of reconstruction success rate, we compare only the reconstruction algorithms used in 2D REmesh.

**Figure 10:**
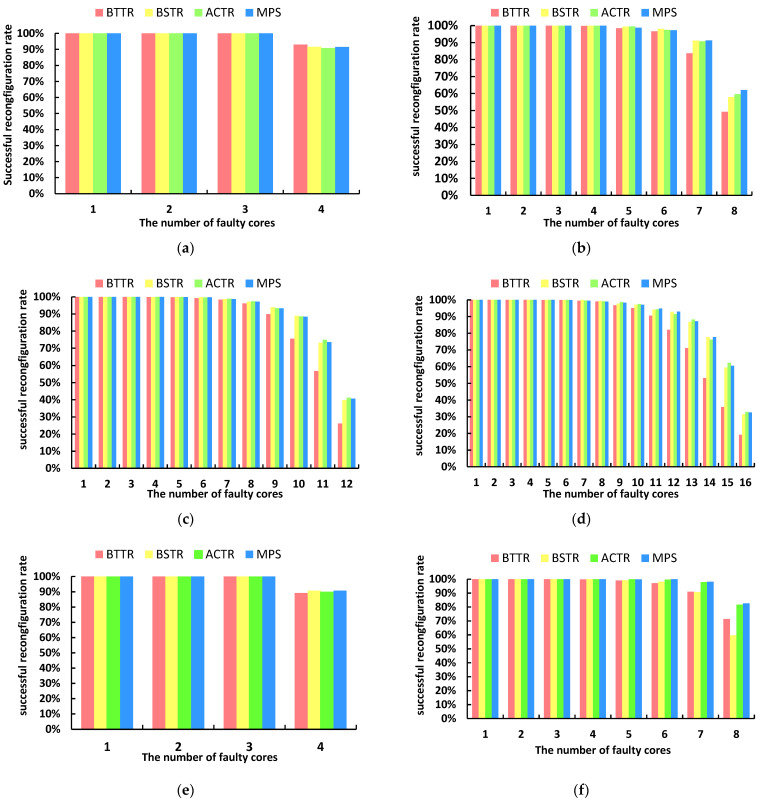
Successful reconfiguration rates of BTTR, BSTR, ACTR, and MPS under different network scales when the number of faulty cores reaches the maximum: (**a**) core scale 4 × 5, (**b**) core scale 8 × 9, (**c**) core scale 12 × 13, (**d**) core scale 16 × 17, (**e**) core scale 4 × 9, (**f**) core scale 8 × 11.

Figure 10 shows the trend in reconstruction success rates for the four algorithms in a 2D REmesh structure as the number of faulty cells increases, across different fault scales. The results indicate that when the number of faulty cells is small, all algorithms achieve a success rate close to 100% regardless of whether the topology is square or non-square. However, as fault density increases, the success rates of all algorithms naturally decline due to the depletion of spare resources and topological isolation. When considering square topologies, although the MPS algorithm employs a local reconstruction strategy to replace faulty cores with as few redundant cores as possible, its introduction of a region-preservation mechanism and a local rollback mechanism enables it to perform well when handling multiple fault conflicts. Even in the large-scale 16 × 17 scenario, its success rate is on par with the high-performing BSTR and ACTR algorithms, exceeding BSTR by 1.1% and falling short of ACTR by 0.3%, both demonstrating relatively high reconstruction success rates; When dealing with non-square topologies, the MPS algorithm slightly outperforms other algorithms in reconstruction success rate. For example, in an 8 × 11 REmesh where the number of failed cores reaches eight, the topological reconstruction success rate of this algorithm reaches 82.60%, which is 0.9% higher than the ACTR algorithm, 11.20% higher than the BTTR algorithm, and 20.90% higher than the BSTR algorithm.

**Figure 11:**
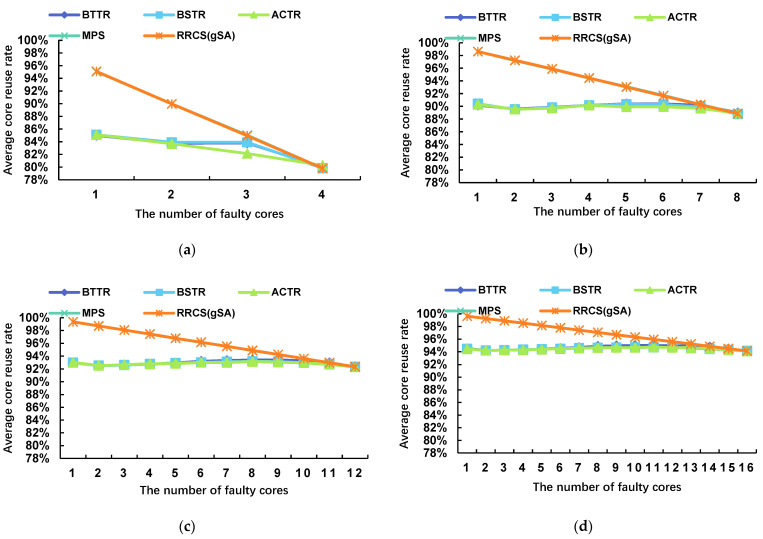

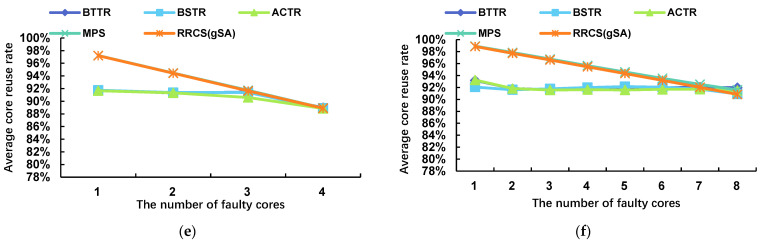
Average core reuse rates of BTTR, BSTR, ACTR, MPS and RRCS(gSA) under different network topologies when the number of faulty cores reaches its maximum: (**a**) core scale 4 × 5, (**b**) core scale 8 × 9, (**c**) core scale 12 × 13, (**d**) core scale 16 × 17, (**e**) core scale 4 × 9, (**f**) core scale 8 × 11.

(2) Average Core Reuse Rate (ACRR):

Figure 11 shows the trend in the average core reuse rate of the four algorithms across different fault scales as the number of faulty cores increases. In both square and non-square topologies, the average core reuse rate of our algorithm is significantly higher than that of the other three comparison algorithms. In particular, in the low-fault-count scenario of an 8 × 9 square topology array, the MPS algorithm achieves an average core utilization rate of over 95%. When the number of faults is one, its average core utilization rate is 98.62%, which is 8.22% higher than that of BSTR, the best-performing algorithm among the others. In an 8 × 11 non-square topology with a single failed core, the average core reuse rate is 98.91%, which is 5.67% higher than that of the best-performing ACTR. Only when the number of failures is high does the average core reuse rate of the MPS algorithm become comparable to that of other algorithms. Additionally, the average core reuse rate of the MPS algorithm exhibits a linear trend across different fault scales, which aligns precisely with our objective of establishing a mapping between faulty cores and redundant cores. Traditional algorithms (such as ACTR) typically employ a global reconstruction strategy; when a faulty core appears in the working area, they attempt to utilize resources from redundant columns as much as possible, resulting in the displacement of nodes across entire rows or columns. In contrast, the MPS algorithm, based on geometric ordering, constructs a compact local shift chain, reducing unnecessary shifts from healthy cells to redundant cells. It successfully limits topological changes to the smallest possible area around the failure point, thereby maximizing the preservation of the original mapping relationship and significantly improving cell reuse rates. Compared to the RRCS(gSA) algorithm, which is based on a 2D mesh structure, the MPS algorithm employs the same principle as RRCS—namely, identifying corresponding redundant kernels to replace defective kernels in the working domain. As shown in the figure, the performance of MPS on ACCR is largely consistent with that of RRCS(gSA); any minor discrepancies are primarily due to instances where the MPS algorithm was unable to successfully reconstruct the image.

**Figure 12:**
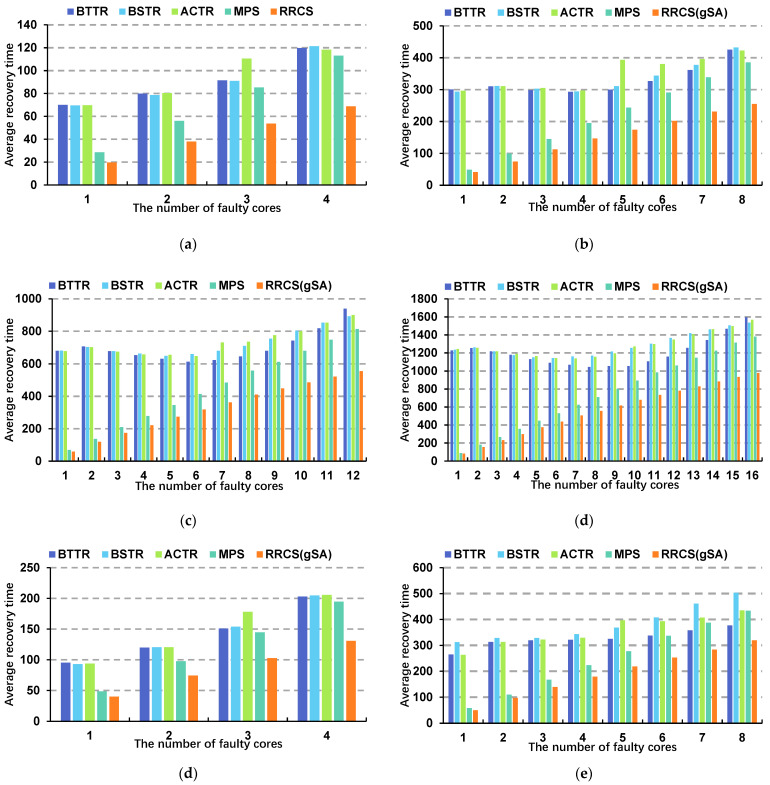
Average recovery times for BTTR, BSTR, ACTR, MPS, and RRCS(gSA) at different network scales when the number of failed cores reaches its maximum: (**a**) core scale 4 × 5, (**b**) core scale 8 × 9, (**c**) core scale 12 × 13, (**d**) core scale 16 × 17, (**e**) core scale 4 × 9, (**f**) core scale 8 × 11.

(3) Average Recovery Time (*ART*):

Figure 12 shows the average recovery times of the four algorithms under different fault scales as the number of faulty cores increases. The MPS algorithm achieved the lowest recovery time across all test groups. The smaller the fault scale, the shorter the recovery time for the MPS algorithm, thanks to the use of a local reconstruction strategy and the construction of monotonic paths. The results show that with a core size of 16 × 17 and a single faulty core, the MPS algorithm’s average recovery time was only 88.68 ns, whereas the BTTR algorithm—which had the shortest recovery time among the other algorithms—had an average recovery time of 1226.74 ns. Even with 16 faulty cores, the MPS algorithm effectively reduced the average recovery time to 1380.44 ns, whereas the BSTR algorithm, which had the shortest average recovery time among the other algorithms, recorded 1536.18 ns—a reduction of 10.14%. Judging by the average recovery times for non-square topologies such as 4 × 9 and 8 × 11, the characteristics of the MPS algorithm are equally applicable in non-square topologies. When the core size is 8 × 11 and there is one failed core, the average recovery time of the MPS algorithm is only 58.04 ns, whereas the best-performing ACTR algorithm requires 263.1 ns. Since the monotonic path constructs the shortest path from the faulty core to the redundant core while minimizing conflicts and reducing the use of unnecessary redundant cores, the overhead associated with data migration can be significantly reduced, ensuring that the system can resume normal operation relatively quickly after a failure occurs. Compared to the RRCS(gSA) algorithm based on a 2D mesh structure, since RRCS(gSA) performs reconstruction of irregular topological structures, during the migration process, it only needs to directly migrate the information from faulty cores to redundant cores in the feasible reconstruction scheme. Therefore, there is no need to perform a global migration within a local region, giving this algorithm a significant advantage in terms of migration time. With a core size of 16 × 17, and 16 faulty cells, the average time taken by RRCS(gSA) for information migration is only 978.52 ns, representing a 29.12% improvement over MPS.

**Figure 13:**
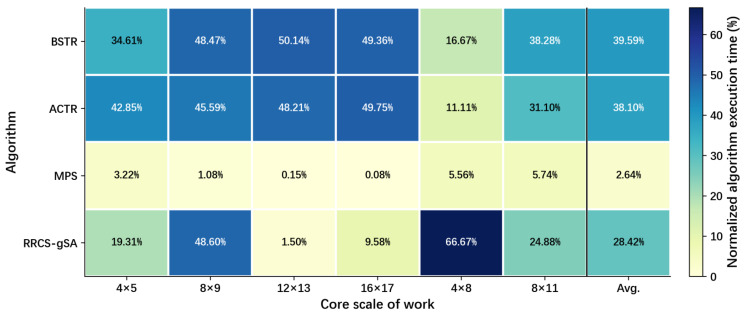
Execution times of the normalized reconstruction algorithms BSTR, ACTR, MPS, and RRCS-gSA in networks with different topologies.

(4) Algorithm Execution Time (AET):

Figure 13 shows a comparison of the normalized execution times for the four topology reconstruction algorithms. In this test, the BTTR algorithm’s heavy use of recursion and pointers resulted in excessive recursion levels, significantly increasing runtime and causing its average recovery time to be notably longer than that of other algorithms. Meanwhile, the RRCS algorithm is overly simple and recovers too quickly; therefore, this metric is compared only among BSTR, ACTR, MPS, and RRCS-gSA, as shown in the figure. The normalized runtime of the algorithms is set to 1.00. In contrast, the average recovery time of our algorithm is significantly better than that of the BSTR and ACTR algorithms, and this advantage becomes even more pronounced in large-scale scenarios. Experimental results show that, compared to the BSTR algorithm, our algorithm reduces average execution time by 36.95%; compared to the ACTR algorithm, the MPS algorithm reduces it by 35.46%; and compared to the RRCS-gSA algorithm, the MPS algorithm reduces it by 25.78%. Therefore, our algorithm achieves a significant advantage in terms of time complexity and can provide a reconstruction scheme in a shorter time.

## 6. Conclusions

This paper proposes a mapping/topology reconstruction method (MPS) based on monotonic path shifting for the core-level redundancy system of 2D REmesh NoCs. The method progressively shifts failed nodes to spare columns using two types of monotonic paths—upward and downward—while employing region retention and local rollback mechanisms to mitigate path intersection conflicts in multi-fault scenarios. While maintaining high reconstruction success rates, MPS generates more compact local shift chains, thereby improving core retention rates and reducing average recovery time. This approach satisfies latency constraints for online or near-online fault recovery, making it highly suitable for deployment in large-scale REmesh NoCs.

## Figures and Tables

**Table 1:** Symbol definition and description.

Symbol Definition	Description
p	Processor core
f	Bad core
F	Bad core collection
V	A collection of processor cores
$\pi_{i}$	The path of the first *i*th bad core structure
S	Path family collections

## Data Availability

The article presents the original contributions of the study. Should further discussion or questions arise, the corresponding author can be contacted directly.
